# Therapeutic applications of a novel humanized monoclonal antibody targeting chemokine receptor CCR9 in pancreatic cancer

**DOI:** 10.1002/1878-0261.70062

**Published:** 2025-05-28

**Authors:** Hannah G. McDonald, Anna M. Reagan, Charles J. Bailey, Mei Gao, Muqiang Gao, Angelica L. Solomon, Michael J. Cavnar, Prakash K. Pandalai, Mautin T. Barry‐Hundeyin, Megan M. Harper, Justin A. Rueckert, Ángela Turrero, Araceli Tobio, Anxo Vidal, Daniel Roca‐Lema, Elia Álvarez‐Coiradas, Pablo Garrido, Laureano Simón, Joseph Kim

**Affiliations:** ^1^ Division of Surgical Oncology, Department of Surgery The University of Kentucky Lexington USA; ^2^ Department of Pathology and Laboratory Medicine The University of Kentucky Lexington USA; ^3^ Cell Cycle & Oncology Group, CiMUS Universidade de Santiago de Compostela Spain; ^4^ SunRock Biopharma, Santiago de Compostela Spain

**Keywords:** chemokine receptor CCR9, immune checkpoint, pancreatic cancer

## Abstract

The relative failure of immune checkpoint inhibitors in pancreatic ductal adenocarcinoma (PDAC) despite having a dense, immunosuppressive tumor microenvironment highlights the need to target alternate/escape pathways. We have previously examined C–C chemokine receptor type 9 (CCR9) as a candidate immune checkpoint and developed a targeted, humanized monoclonal antibody (SRB2). Cytotoxicity of SRB2 was evaluated *in vitro* and *in vivo*. CCR9 expression on PDAC cells/tissues, immune components of patient‐derived organoids (PDOs), and antibody‐dependent cell‐mediated cytotoxicity were examined. In PANC‐1 and MIA PaCa‐2 cell lines, we demonstrated highest CCR9 expression; however, no direct cytotoxic effect was observed with SRB2 treatment. In PANC‐1 cells, NK cell‐mediated cytotoxicity was promoted by SRB2. Dose‐dependent SRB2 cytotoxicity was observed in PDAC PDOs. In patient‐derived xenograft mouse models, cytotoxicity of SRB2 monotherapy and in combination with oxaliplatin was also shown. In humanized immune‐competent mouse models, SRB2 efficacy was similar to other drugs, but two mice in this cohort had complete tumor regression. Our current studies suggest that therapeutic targeting of CCR9 may improve PDAC outcomes, and additional studies are underway to evaluate SRB2 for clinical use.

## Graphical abstract

C–C chemokine receptor type 9 (CCR9) is an immune checkpoint in pancreatic ductal adenocarcinoma (PDAC). Novel anti‐CCR9 antibody SRB2 was evaluated in combination with cytotoxic chemotherapy in PDAC cells, patient‐derived organoids, patient‐derived xenografts, and humanized mouse models. SRB2 may improve outcomes in patients with PDAC when used in combination with cytotoxic chemotherapy.

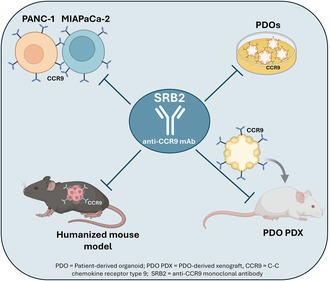

**Abbreviations**
ADCC: antibody‐dependent cellular cytotoxicity
ATCC: American Type Culture Collection
BME: basement membrane extract
CCL25: chemokine ligand 25
CCR9: C–C chemokine receptor type 9
cDNA: complementary DNA
FBS: fetal bovine serum
GAPDH: glyceraldehyde‐3‐phosphate dehydrogenase
GEPIA: Gene Expression Profiling Interactive Analysis
GPCR: G‐protein coupled receptor
GTEx: Genotype‐Tissue Expression
H&E: hematoxylin and eosin
HRP: horseradish peroxidase
ICIs: immune checkpoint inhibitors
IF: Immunofluorescence
IgG1: immunoglobulin G1
IHC: immunohistochemical
IO: immune oncology
mAB: monoclonal antibody
NCG: NOD‐Prkdc^em26Cd52^Il2rgem^26Cd22^/NjuCrl
NK: natural killer
PBS: phosphate‐buffered saline
PCR: polymerase chain reaction
PD1: programed cell death protein 1
PDAC: pancreatic cancer
PDL1: Programed cell death ligand 1
PDO: patient‐derived organoid
PDX: patient‐derived xenograft
qPCR: quantitative real‐time polymerase chain reaction
SD: standard deviation
SEM: standard error of the mean
TCGA: The Cancer Genome Atlas
TME: tumor microenvironment
V: volume

## 1 Introduction

Pancreatic duct cancers have a dense stromal tumor microenvironment (TME) that promotes tumor growth, survival, and immune evasion. Immune oncology (IO) therapies such as immune checkpoint inhibitors (ICIs) target immune‐evasive pathways in the TME to bolster T‐cell‐mediated cytotoxicity [1]. IO drugs, such as anti‐programed cell death protein 1 (PD1) or anti‐programed cell death ligand 1 (PDL1) drugs, are approved in multiple different cancer types (e.g., kidney, liver, bladder, and lung cancers) [2], yet they have failed to change the therapeutic landscape for patients with pancreatic ductal adenocarcinoma (PDAC) [3]. In our efforts to better understand how to improve targeting of the PDAC TME with IO agents, we identified C–C chemokine receptor type 9 (CCR9) as a candidate immune checkpoint. Here, we tested the therapeutic efficacy of SRB2, a humanized immunoglobulin G1 (IgG1) monoclonal antibody (mAb) with an optimized Fc that triggers enhanced antibody‐dependent cell‐mediated cytotoxicity (ADCC), which was developed to target CCR9.

CCR9 is a G‐protein coupled receptor (GPCR) responsible for the development and maturation of T cells. The CCR9/chemokine ligand 25 (CCL25) chemokine receptor‐ligand axis is not only responsible for T‐cell development and immune cell recruitment, but also appears to support the invasive phenotype of solid tumors [4, 5, 6]. In fact, our group previously investigated CCR9, showing its high expression levels in PDAC tissues and its regulation of PDAC growth and proliferation [5]. Notably, we discovered that the ligand CCL25 was secreted by pancreatic stellate cells in the PDAC TME, suggesting pro‐survival and anti‐immune paracrine signaling pathways [5]. More recently, we characterized novel CCR9‐mediated signaling that promotes PDAC progression through the activation of β‐catenin [7].

Due to the CCR9 roles in regulating T‐cell development and promoting PDAC invasiveness, we hypothesized that CCR9 functions as an immune checkpoint that attenuates T cells and facilitates evasion of immune‐mediated cancer cell death. To explore therapeutic antagonism of CCR9 and its candidacy as an immune checkpoint, we tested the therapeutic activity of a novel anti‐CCR9 therapeutic mAb SRB2 (SunRock Biopharma, Spain) *in vitro* and *in vivo*. This mAb has a high binding affinity to CCR9 and has demonstrated potent ADCC via natural killer (NK) cells *in vivo* [8]. We hypothesized that targeting CCR9 with SRB2 may be a novel therapeutic approach for patients with PDAC.

## 2 Materials and methods

### 2.1 Cell culture

The established PDAC cell lines AsPC‐1 (RRID: CVCL_0152), CaPan‐2 (RRID: CVCL_0026), MIAPaCa‐2 (RRID: CVCL_0428), and PANC‐1 (RRID: CVCL_0480) were obtained from the American Type Culture Collection (ATCC). The identities of these cell lines were verified using the ExPASy Cellosaurus database. All cell lines were authenticated within the past 3 years through short tandem repeat profiling. Additionally, all cell lines were routinely tested for Mycoplasma contamination using a polymerase chain reaction (PCR)‐based assay, and only Mycoplasma‐free cells were used for experiments. To minimize genetic drift, all experiments were conducted using early passage cells (i.e., < *P*10). Given the time elapsed between receiving P0 cells from ATCC, cell banking, and subsequent use, we further validated the genomic integrity of our cell lines through ATCC to confirm their identity and ensure reliability.

MIAPaCa‐2 and PANC‐1 cells were cultured in Dulbecco's Modified Eagle Medium (Gibco) supplemented with 10% fetal bovine serum (FBS), 1% Penicillin–Streptomycin (10 000 IU/mL), and 1% L‐glutamine (200 mM) (Thermo Fisher, Waltham, MA, USA). RPMI 1640 media (Gibco, Waltham, MA, USA) supplemented with 10% FBS was used for AsPC‐1 cells. McCoy's 5A medium (ATCC, Manassass, VA, USA) supplemented with 10% FBS was used for Capan‐2 cells. All cell lines were maintained at 37 °C in a humidified atmosphere at 5% CO_2_. Cell lines were passaged every 3–4 days at 70–80% confluence.

### 2.2 Development of patient‐derived organoids

Patient‐derived organoids (PDOs) were generated as previously described [9]. Briefly, PDAC tissues were minced, digested, and washed. The cell pellet was resuspended in reduced growth factor basement membrane extract (BME; Trevigen, Minneapolis, MN, USA) and cultured in complete PDO medium supplemented with Y27632 (10 μM). Culture medium was exchanged every 2–3 days, and PDOs were passaged every 5–7 days when 70–80% confluent.

### 2.3 Development of PDO single cells for drug testing

Following growth and passaging, PDOs were dissociated into single cells for drug testing using previously described methodology [9, 10]. In brief, PDOs were dissociated into single cells and plated in 96‐well plates at 2000–5000 cells/10 μL/well in 50% BME. After approximately 3 days, once PDO single cells formed small PDOs, drug testing was initiated.

### 2.4 RNA extraction, reverse transcription, and quantitative real‐time PCR

PDAC cells and tissues were homogenized with cold phosphate‐buffered saline (PBS) and centrifuged for collection. Cell or tissue pellets were stored at −80 °C before RNA extraction. Total RNA was extracted using the RNeasy Plus Mini Kit (Qiagen, Germantown, MD, USA). Reverse transcription was performed using a complementary DNA (cDNA) Kit (Applied Biosystems) on a C1000 Touch Thermal Cycler (Bio‐Rad). Quantitative real‐time PCR (qPCR) was performed on the QuantStudio 3 Real‐Time PCR Machine (Applied Biosystems) using standardized thermal cycling conditions: 50 °C for 2 min, 95 °C for 10 min, followed by 40 cycles of 95 °C for 15 s and 60 °C for 1 min. Primers of human glyceraldehyde‐3‐phosphate dehydrogenase (GAPDH) and CCR9 were synthesized (Integrated DNA Technologies) with the following sequences: GAPDH (102 bp), forward 5′‐CAAGAGCACAAGAGGAAGAGAG‐3′ and reverse 5′‐CTACATGGCAACTGTGAGGAG‐3′; and CCR9 (166 bp), forward 5′‐ACACCCACAGACTTCACAAG‐3′ and reverse 5′‐AGCCAGTACAAGGGTGGGA‐′ All PCR assays were run in duplicate and for at least 2 independent experiments. *C*_t_ values were graphed to show the relative expression levels across samples.

### 2.5 Western blot assays

Western blot assays were performed to evaluate CCR9 protein expression in PDAC cells and PDOs as described [11]. Blots were incubated overnight at 4 °C with rabbit primary antibodies against CCR9 (1 : 1000, Invitrogen) or mouse primary antibody against β‐actin (1 : 5000; Sigma‐Aldrich, St. Louis, MO, USA) for loading control. Then the blots were incubated with a corresponding horseradish peroxidase (HRP)‐conjugated secondary antibody (1 : 5000; Santa Cruz Biotechnology, Dallas, TX, USA), visualized in enhanced chemiluminescence solution (SuperSignal West Pico Chemiluminescent Substrate; Thermo Fisher) and exposed with an UVP ChemiDoc‐It2imager. Band intensity was quantified using ImageJ (NIH) and results analyzed using GraphPad Prism software (v9).

### 2.6 CCR9 expression from The Cancer Genome Atlas

GEPIA (Gene Expression Profiling Interactive Analysis, http://gepia.cancer‐pku.cn/index.html) was used to graphically display CCR9 gene expression in PDAC and matched normal clinical specimens. Data in GEPIA was extracted from The Cancer Genome Atlas (TCGA) and Genotype‐Tissue Expression (GTEx) Project. CCR9 gene expression was displayed in log_2_ (TPM+1) for log‐scale in the form of a box plot for visualization.

### 2.7 Immunofluorescence staining

Immunofluorescence (IF) staining was performed to evaluate CCR9 expression and localization of CCR9 in PDOs and patient‐derived xenograft (PDX) tissues as described [10, 11]. Primary antibodies used to stain tissue were mouse anti‐PD1 (1 : 150; Cell Marque, Rocklin, CA, USA), rabbit anti‐CD3 (1 : 100; Proteintech), along with secondary antibodies goat anti‐mouse Alexa Fluor 555 (1 : 500; Abcam, Waltham, MA, USA) and goat anti‐rabbit Alexa Fluor 488 (1 : 500; Invitrogen). Primary antibodies used to stain PDOs were rabbit anti‐CD3 (1 : 100; Proteintech, Rosemont, IL, USA) and rat anti‐CK19 (1 : 100; DSHB, Iowa City, IA, USA), along with secondary antibodies goat anti‐rabbit Alexa Fluor 647 (1 : 500; Abcam) and goat anti‐rat Alexa Fluor 488 (1 : 500; Abcam). Secondary antibodies alone were used as antibody controls. VectaShield with DAPI (Vector Laboratories) was applied to the stained cells and cells were imaged using a Nikon Ts2 confocal microscope.

### 2.8 Flow cytometric analysis

Cell surface CCR9 expression was assessed by flow cytometry assays as described [11]. PDAC cells were plated on 100 mm^3^ culture dishes and dissociated with trypsin‐ethylenediaminetetraacetic acid (Gibco). Cells were probed with primary antibody rabbit anti‐CCR9 (1 : 100; Invitrogen) and conjugated antibody mouse anti‐CD45 APC/Cy7 (1 : 100; BioLegend, San Diego, CA, USA) for 1 h in flow buffer (PBS/0.1% BSA) at 4 °C. Cells were washed and additionally incubated with mouse anti‐rabbit AlexaFlour 647 fluorescent secondary antibody (1 : 500; Abcam) for 1 h at room temperature (RT). Cells were then fixed in 2% paraformaldehyde. Flow cytometric analysis was performed on a BD FACSymphony A3 (BD Sciences). FlowJo™ software (v10) was used for data analysis.

### 2.9 Cell proliferation assays

PDAC cells were plated in clear 96‐well plates at 5000 cells/100 μL/well and allowed to attach overnight before treatment. PDOs were dissociated into single cells as described [9] and plated at 500–3000 cells/10 μL/well in 96‐well clear bottom white plates for viability analysis. Cells were treated with IgG1 isotype antibody control, SRB2 (1–100 μg/mL; SunRock Biopharma, Santiago de Compostela, Spain), oxaliplatin (0–200 μg/mL; LC Labs), irinotecan (0–200 μg/mL; LC Labs), or paclitaxel (0–200 μg/mL; LC Labs, Woburn, MA, USA) at 8 concentrations in triplicate for 48 h. The chemotherapy drugs (oxaliplatin, irinotecan, and paclitaxel) were selected for testing since they are the components of standard‐of‐care PDAC chemotherapy regimens (FOLFIRINOX and gemcitabine/nab‐paclitaxel) [12].

Cell viability was measured using the WST‐1 reagent (Roche) for cells and CellTiter‐Glo® 3D Cell Viability Assay (Promega, Madison, WI, USA) for PDOs. Dose–response curves were graphed with GraphPad Prism software (v9) and IC_50_ values were calculated. The approximate IC_50_ dose of oxaliplatin in each PDO line was used for subsequent testing in combination with SRB2.

### 2.10 ADCC assay in PDAC cells

Blood from healthy donors was extracted and NK cells were then isolated using the NK cells Isolation Kit (Miltenyi Biotec, Gaithersburg, MD, USA). Purified NK cells were analyzed for CD3 and CD56 expression by flow cytometry, and preparations of at least 70% purity were used for subsequent assays.

For cytotoxicity assays, PANC‐1 cells were pre‐incubated for 30 min with 10 μg/mL of either SRB2 (SunRock Biopharma) or isotype control. Then, cells were co‐cultured at a 20 : 1 ratio (NK : tumoral cell) with the isolated human NK cells for 4 h. To identify the CCR9^+^ population, cells were stained with conjugated anti‐CCR9‐phycoerythrin (Thermo Fisher) for 30 min at RT, then stained with live/dead dye Aqua (Invitrogen) for 15 min and analyzed with flow cytometry (FlowJo™ software v10). CCR9^+^ and CCR9^−^ cells were identified and cell death was determined for both populations by Aqua staining. Specific cell lysis for each population was calculated as 100 × [(% dead target cells in sample with anti‐CCR9 mAb – % dead target cells in sample with isotype)/(100 – % dead target cells in sample with isotype)].

### 2.11 *In vivo* drug efficacy

A human PDAC treatment‐naïve operative tumor specimen (hPT15) was xenografted into NOD‐SCID‐gamma mice (Jackson Laboratory, Gaithersburg, MD, USA) as described [10, 13]. These PDX tumors were expanded and subsequently implanted as 20–30 mm^3^ sections into 48 mice. When tumor size reached ~100 mm^3^ 2–3 weeks after implantation, mice were randomly divided into six treatment groups: solvent (negative control), Herceptin (10 mg/kg; Genentech, mAb treatment control), SRB2 (20 mg/kg, SunRock Biopharma), oxaliplatin (10 mg/kg, Apotex, Weston, FL, USA), oxaliplatin + SRB2, and oxaliplatin + Herceptin. Mice were treated with intraperitoneal injections once weekly. Mice were weighed and tumor size was measured twice weekly by digital caliper for 4 weeks.

NOD/Shi‐scid/IL‐2Rγnull (NCG) mice, a novel immunodeficient mouse strain recently developed for IO applications, were used as the humanized mouse models [14, 15]. NCG mice were transplanted with CD34^+^ hematopoietic stem cells isolated from human cord blood after a chemoablation treatment. Complete hematopoiesis was fully recapitulated, and 14 weeks after CD34^+^ engraftment, a fully functional human immune system was established. First, PDAC PDOs (i.e., hPT15) were amplified into five immunodeficient stock mice. Once tumor volumes reached 1000 mm^3^, tumors were harvested, cut into small portions, and implanted subcutaneously into the humanized NCG mice. Once average tumor volumes reached 101–105 mm^3^, mice were randomized into four treatment groups according to their tumor volume, cord blood donor, and humanization rate: vehicle (negative control), SRB2 (25 mg/kg Q7D), pembrolizumab (8 mg/kg Q3D) and gemcitabine + nab‐paclitaxel (50 and 5 mg/kg Q7D, respectively). Mice were monitored for weight and health. Tumor volume was measured twice/thrice per week using a caliper.

Tumor volume (*V*) was calculated using the formula V = 1/2(length×width^2^). Relative tumor volumes were compared between treatment groups, where relative tumor volume equaled the tumor volume at study time points relative to the initial tumor volume.

### 2.12 Histology and immunohistochemistry analysis

Excised PDX tumors were fixed in 10% formalin and submitted to the University of Kentucky Biospecimen Procurement and Translational Pathology Shared Resource Facility for histological sectioning and immunohistochemical (IHC) staining as described [16]. Primary antibodies against Ki‐67 (1 : 100; Abcam) and cleaved caspase‐3 (CC3, 1 : 150; Cell Signaling) were incubated at 37 °C for 1 h followed by detection with Ventana OmniMap anti‐rabbit‐HRP (Roche) and ChromoMap DAB (Roche, Weston, FL, USA). To quantify tumor necrosis and proliferation, the hematoxylin and eosin (H&E)‐stained slides were first evaluated for tumor, and the percent of non‐viable tumor tissue was estimated for applicable cases. The Ki‐67 immunostained sections were examined to estimate the overall proliferative index of the entire tumor on the microscopic slide.

### 2.13 Statistical analysis

Results from independent experiments are presented as mean ± standard deviation (SD) or standard error of the mean (SEM), as specified. For two‐group analysis, Student's *t*‐tests were used. Two‐way ANOVA was used for multigroup comparisons unless otherwise specified. Mixed‐effects analysis was used for PDX data due to incomplete data secondary to early mouse demise. Specifically, one of eight mice in two different treatment groups died prior to the final time point. Analyses of the humanized animal studies presented by treatment group were performed using a one‐way ANOVA to evaluate differences between treatment groups, followed by Tukey's *post hoc* test for multiple comparisons. Differences were considered significant at *P* < 0.05. Statistical analyses were performed using GraphPad Prism software (v9).

### 2.14 Ethics approval

This work has been carried out in accordance with Ethical Principles for Medical Research Involving Human Subjects (i.e., World Medical Association Declaration of Helsinki). Written informed consent was obtained from pancreatic cancer patients for procurement of surgical tumor specimens. PDAC tissue analysis/PDO generation and subsequent analysis was conducted under the authorization of the University of Kentucky Medical Institutional Review Board for this study (Protocol #48495). For ADCC assays, written informed consent was obtained from healthy blood donors by the Servizo de Vixilancia da Saude, University of Santiago de Compostela (Spain), under the authorization of the Comité Territorial de Ética de la Investigación de Santiago‐Lugo for this study (Protocol #2019/054).

PDX experiments were conducted as a University of Kentucky Institutional Animal Care and Use Committee‐approved study (Protocol #2018‐3116). Humanized mouse experiments were conducted under the approval of the local ethics committee for animal experimentation (CELEAG) and validated by the French Ministry of Research (Approval number CELEAG APAFIS#38383).

## 3 Results

### 3.1 CCR9 expression in PDAC cells and tissues

We evaluated CCR9 expression in PDAC cells and tissues using qPCR and western blot. Among 4 PDAC cell lines (CAPAN‐2, AsPC‐1, MIAPaCa‐2, and PANC‐1), MIAPaCa‐2 and PANC‐1 had the highest CCR9 protein and RNA levels (Figs S1 and S2). Our results showed varied CCR9 expression levels among clinical specimens, with hPT15 PDO displaying the highest CCR9 expression (Fig. S1). To corroborate our findings, we utilized TCGA and observed heterogeneous CCR9 expression levels in PDAC clinical specimens (*N* = 179) compared to generally low levels in matched normal tissues (*N* = 171) (Fig. S3).

### 3.2 SRB2 treatment of PDAC cells

**Fig. 1:**
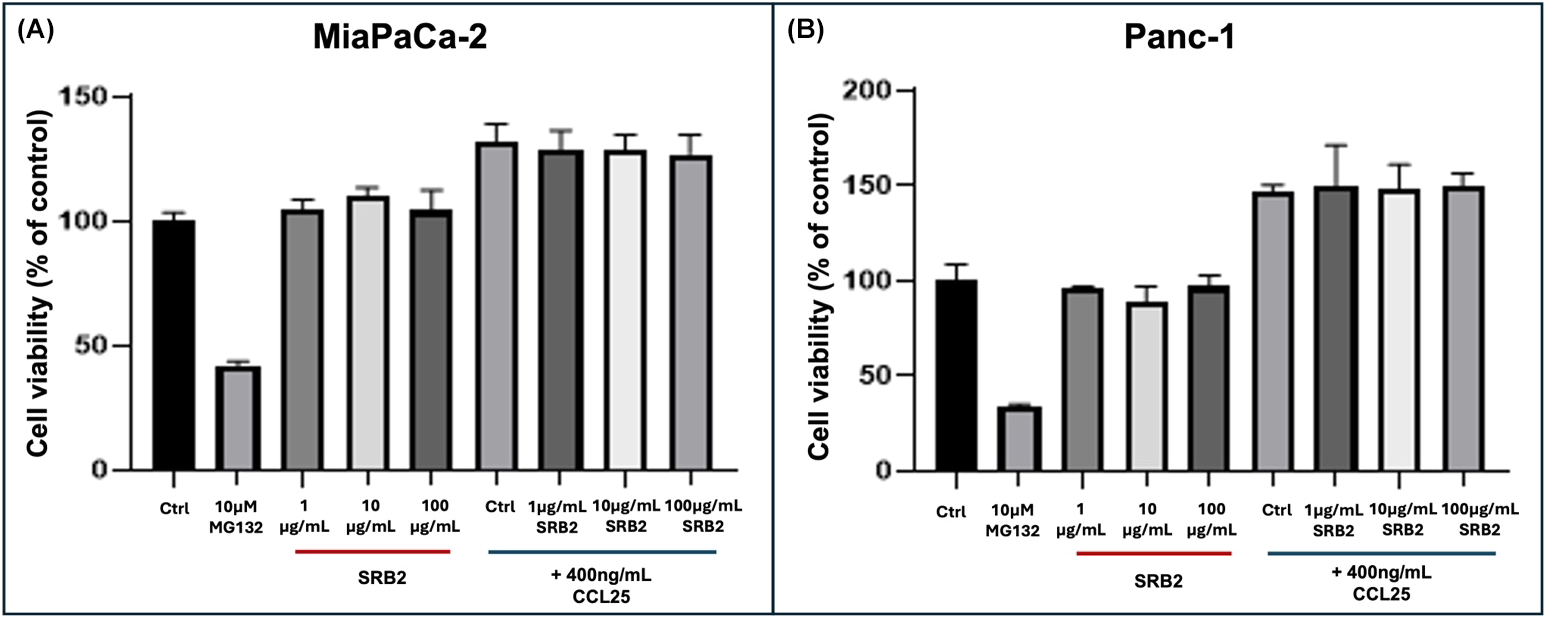
SRB2 and PDAC cell proliferation. The results of this assay demonstrated that SRB2 had no effect on CCL25‐induced PDAC cell proliferation. CCL25 (400 ng/mL) induced MIAPaCa‐2 (A) and PANC‐1 (B) cell proliferation, while SRB2 did not attenuate proliferation in either cell line. MG132 (10 μM) was used as a positive cytotoxic control. There was one biological replicate per group, and data are presented as mean ± SEM.

Based on detection of CCR9 in PDAC cells, we tested the direct effects of SRB2 exposure on CCR9^+^ MIAPaCa‐2 and PANC‐1 cells. Cultured PDAC cells were maintained in serum‐free formula containing solvent control (0.1% BSA) or CCL25 (400 ng/mL) for 5 days. MG132 (10 μM) was used as a cytotoxic positive control. WST‐1 reagent (Roche) was used to measure CCL25‐mediated cell proliferation. Our results showed that SRB2 (1, 10, 100 μg/mL) had no direct cytotoxicity on either PDAC cell line (Fig. 1).

### 3.3 ADCC assay in PDAC cell lines

**Fig. 2:**
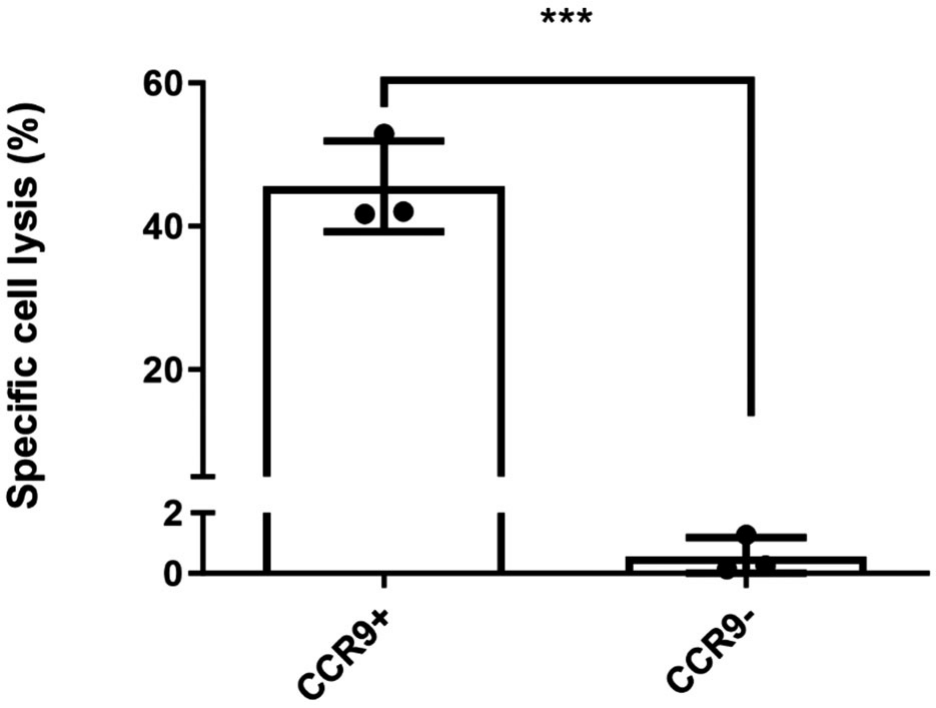
ADCC assay in PANC‐1 cells. NK cell‐mediated cytotoxicity was promoted by SRB2. PANC‐1 cell lysis was measured after treatment with SRB2 (10 μg/mL) and NK cell co‐culture. The results showed significantly higher cell lysis in CCR9^+^ cells compared to CCR9^−^ cells. There were 3 biological replicates included, and data is presented as mean ± SEM and compared with an unpaired Student's *t*‐test.

We tested an effector function (i.e., ADCC) of SRB2. These effector functions are mediated by the Fc domain of the antibody. We observed that the anti‐CCR9 antibody significantly induced cell lysis in CCR9^+^ PANC‐1 cells, whereas it did not affect cell viability in CCR9^−^ cells (Fig. 2).

The results of the ADCC assay demonstrated that when SRB2 (10 μg/mL)‐treated PANC‐1 cells were exposed to NK cells for 4 h, NK cell‐mediated cytotoxicity targeted the CCR9^+^ population in PANC‐1 cells, whereas the CCR9^−^ population was mostly unaffected. Specifically, anti‐CCR9 treatment induced 45.54 ± 6.35% and 0.56 ± 0.062% cell lysis in CCR9^+^ and CCR9^−^ cells, respectively (Fig. 2). Furthermore, no cytotoxic effect was observed in the absence of NK cells, indicating the role of these effector cells in SRB2‐based immunotherapy.

### 3.4 SRB2 cytotoxicity in PDOs and PDXs

**Fig. 3:**
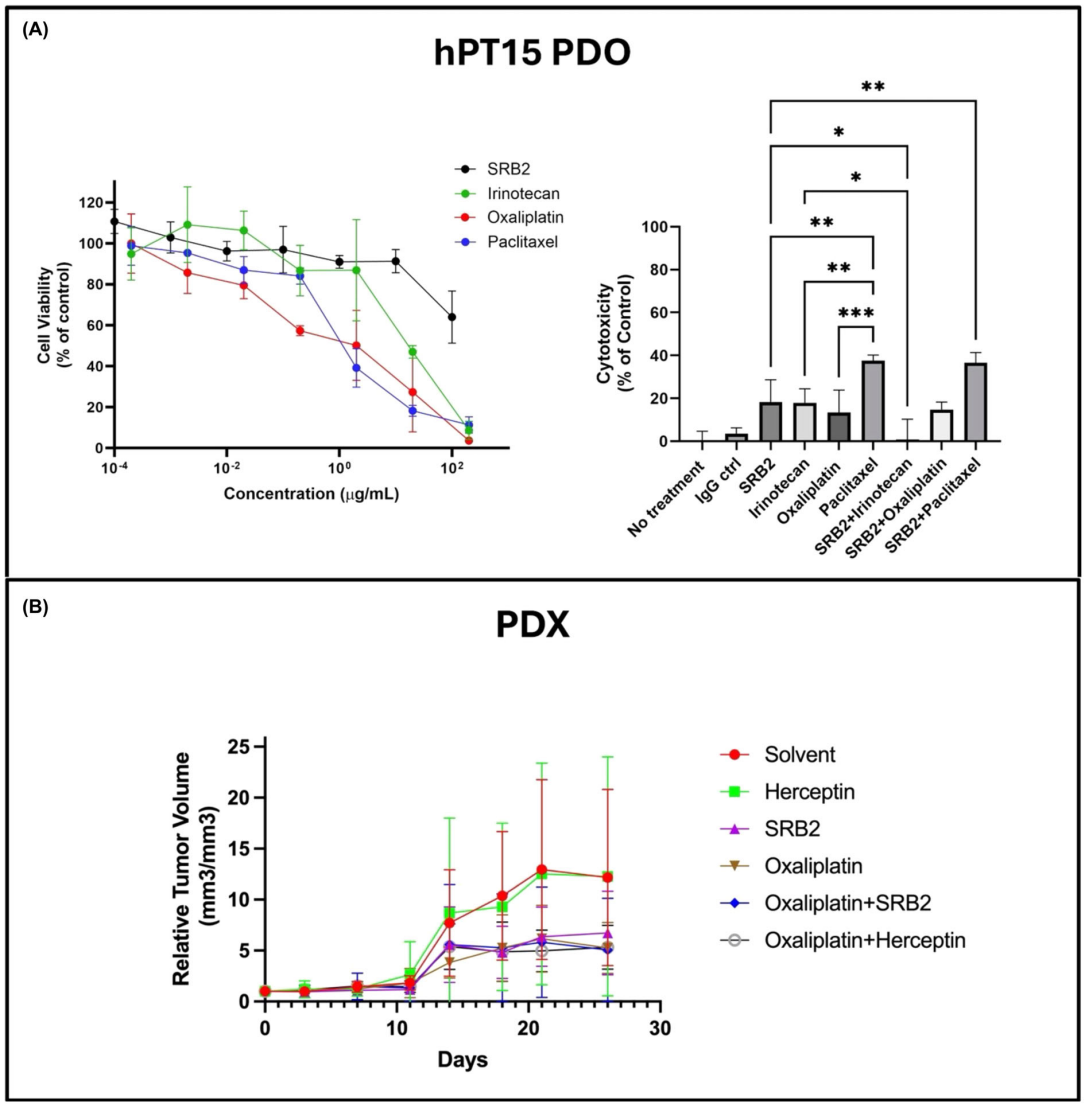
*In vitro* and *in vivo* effects of SRB2. Here, SRB2 was administered alone and in combination with cytotoxic drugs in PDAC PDOs and PDX animals. Dose–response curves of SRB2, irinotecan, oxaliplatin, and paclitaxel are shown for hPT15 PDOs (A). Data include three biological replicates and are presented as mean ± SD. The bar graph shows the effects of SRB2 and/or cytotoxic drugs (*n* = 4 biological replicates per treatment group) (A). Data are presented as mean ± SD and compared using two‐way ANOVA. Then, the effects of SRB2 and cytotoxic/targeted drugs were evaluated in CCR9^+^ PDAC PDX animals. We observed trends toward decreased tumor growth in the experimental treatment groups compared with the control groups (B). *N* = 8 mice were tested per treatment group. PDX data are presented as mean ± SD and compared using mixed‐effects analysis.

We tested one early passage PDO line with SRB2 (0–100 μg/mL), oxaliplatin (0–200 μg/mL), irinotecan (0–200 μg/mL), or paclitaxel (0–200 μg/mL) for 48 h. The results showed that SRB2 cytotoxicity in the PDO line was dose‐dependent with the highest cytotoxicity between 10 and 100 μg/mL (Fig. 3A). The approximate IC_50_ of each drug was subsequently used in treatment combinations with SRB2. SRB2 demonstrated similar cytotoxicity to irinotecan and oxaliplatin (both *P* > 0.05). Paclitaxel demonstrated the highest cytotoxicity, which was statistically significant compared to the other treatments. Cytotoxicity was lower for SRB2 + irinotecan compared to irinotecan alone (*P* = 0.02) but was similar for the SRB2 combination with oxaliplatin and paclitaxel when compared to the individual drugs (both *P* > 0.05).

To substantiate the results of the *in vitro* studies, we evaluated the effects of SRB2 in PDAC PDX animals. We selected hPT15 PDOs for xenografting because this line had the highest CCR9 expression from our qPCR and western blot data (Fig. S1). The results of the *in vivo* study showed that SRB2‐treated mice had decreased tumor growth compared to control groups and similar cytotoxicity to oxaliplatin. While tumor growth rates and excised tumor volumes were not statistically different between the four treatment groups (SRB2, oxaliplatin, SRB2 + oxaliplatin, and oxaliplatin + Herceptin; *P* > 0.05), SRB2 + oxaliplatin had the lowest tumor growth overall (Fig. 3B).

Following volumetric studies, tumors were fixed and processed for H&E and IHC. Sections from the tumors showed similar morphologic findings consisting of a carcinoma with marked squamous differentiation, focal glandular differentiation in most tumors, and varying degrees of non‐viable tumor.

**Fig. 4:**
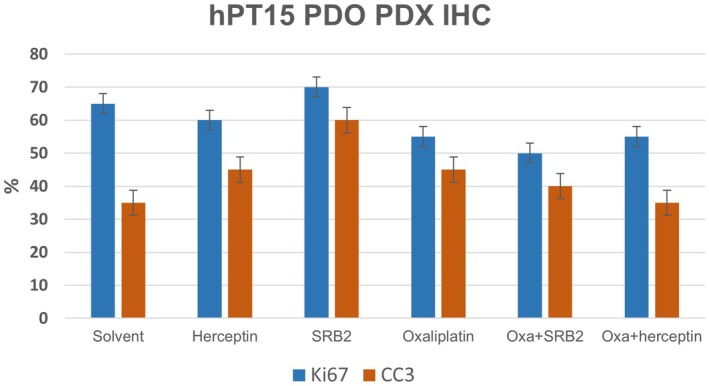
IHC measurements of proliferation (Ki‐67) and necrosis (CC3). Excised tumors from two mice per treatment group (total *n* = 12) were sectioned and IHC analysis performed for Ki‐67 (blue) and CC3 (orange). The results showed the highest necrosis (CC3) with SRB2 and the lowest proliferation (Ki‐67) with SRB2 + oxaliplatin. Data shown as mean ± SEM.

Tumor proliferation (Ki‐67) and necrosis (CC3) were evaluated following IHC staining using % estimates of positive staining compared to total tumor. The CC3 positive immunostained sections correlated approximately with the percent of non‐viable tumor noted on the H&E‐stained sections. The greatest abundance of Ki‐67 was found in the solvent and SRB2 groups. The lowest Ki‐67 was found in the oxaliplatin + SRB2 group. Similarly, the lowest CC3 was observed in the solvent and oxaliplatin + herceptin groups, while the highest CC3 was in the SRB2 group. With the combination of Ki‐67 and CC3, the combined lowest proliferation (Ki‐67) and highest % necrosis (CC3) were observed in the oxaliplatin + SRB2 group, followed by oxaliplatin alone (Fig. 4).

### 3.5 Immune cell components of early passage PDOs and PDXs

**Fig. 5:**
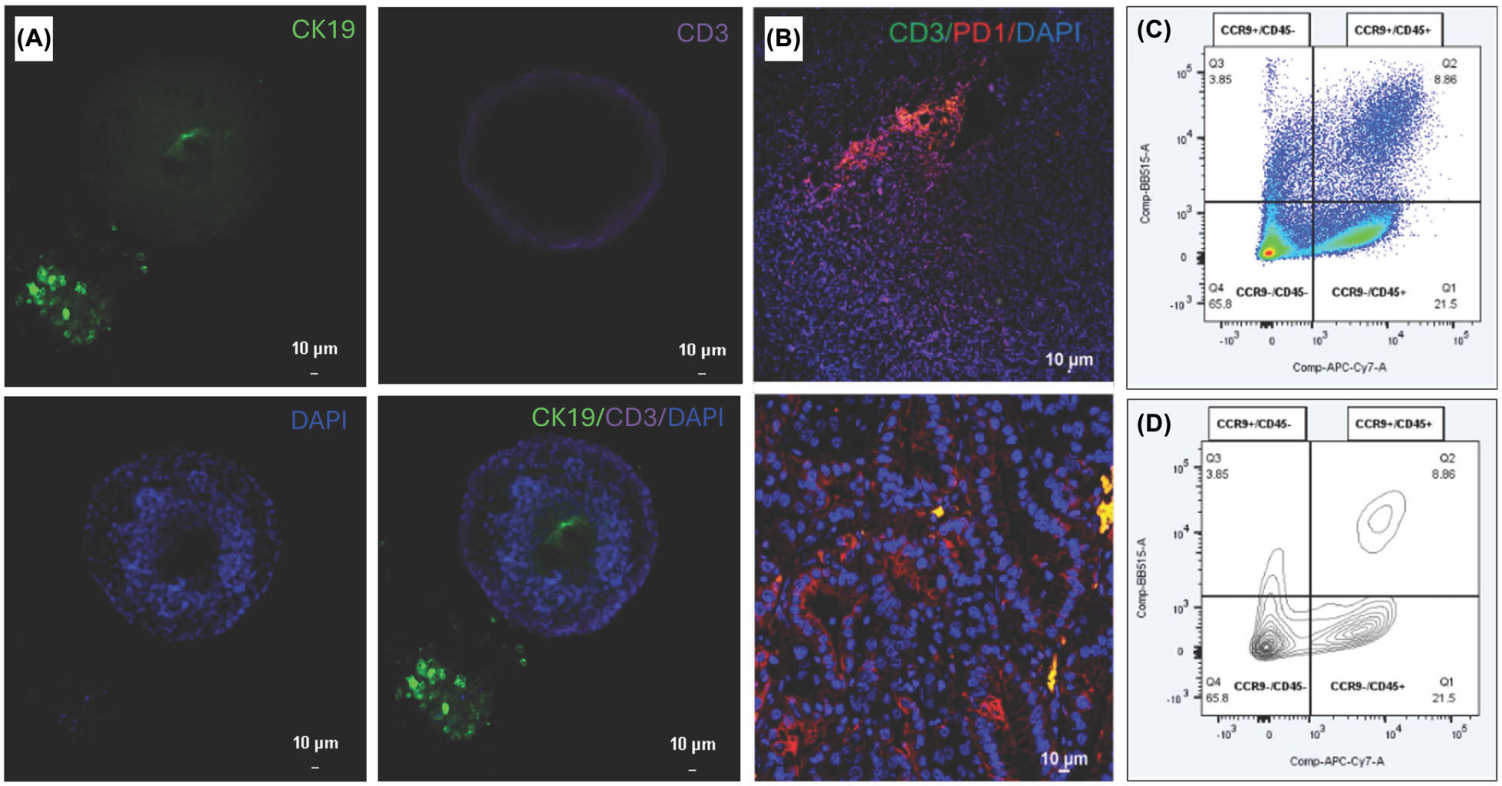
Immune cell populations in PDAC PDOs and PDXs. (A) IF staining for CK19 (green) and CD3 (red) in hPT15 PDOs showed retained immune and cancer cells in early passage PDOs. (B) IF staining for CD3 and PD1 in hPT15 PDX tissue slides showed staining for CD3 (green) and PD1 (red). Overlay (yellow) of both markers indicate immune cells. Scale bar in A and B represents 10 μm. Pseudocolor dot plot (C) and contour plot (D) show proportions of CCR9 and CD45 single positive (Q1,3) and double positive (Q2), as well as double negative (Q4) populations within the tumors. Flow cytometry was performed using BD FACSymphony A3 machine and analyzed using FlowJo v10 Software.

We sought to identify heterogeneous cell populations in PDOs and PDXs, especially in early passages (< 4). Our results revealed retained T‐cell populations (CD3 in purple) in hPT15 early passage PDOs (Fig. 5A). With IF staining of PDX tissues, we observed PD1^+^/CD3^+^ cells (yellow), suggesting T‐cell populations (Fig. 5B). With flow cytometry, we detected 30.4% CD45^+^ and 12.7% CCR9^+^ cells in tissues (Fig. 5C,D). Furthermore, CCR9 expression was noted on both CD45^+^ (8.86%) and CD45^−^ (3.85%) PDX PDO cell populations, suggesting CCR9 expression on both infiltrating lymphocytes and cancer cells in the PDX.

### 3.6 Effect of SRB2 in NCG humanized animals

Having demonstrated the efficacy of SRB2 with an *in vivo* model and described its effector functions, SRB2 was tested in a mouse model with a fully intact humanized immune system (i.e., NCG humanized mice). The study was designed to evaluate the efficacy of SRB2 with standard‐of‐care treatment for locally advanced and metastatic pancreatic cancer (gemcitabine + nab‐paclitaxel) or immunotherapy (pembrolizumab) in humanized mice engrafted with PDAC tumors. We chose the same PDAC hPT15 with high CCR9 expression to further validate the purported mechanism of action of SRB2 in this *in vivo* model.

**Fig. 6:**
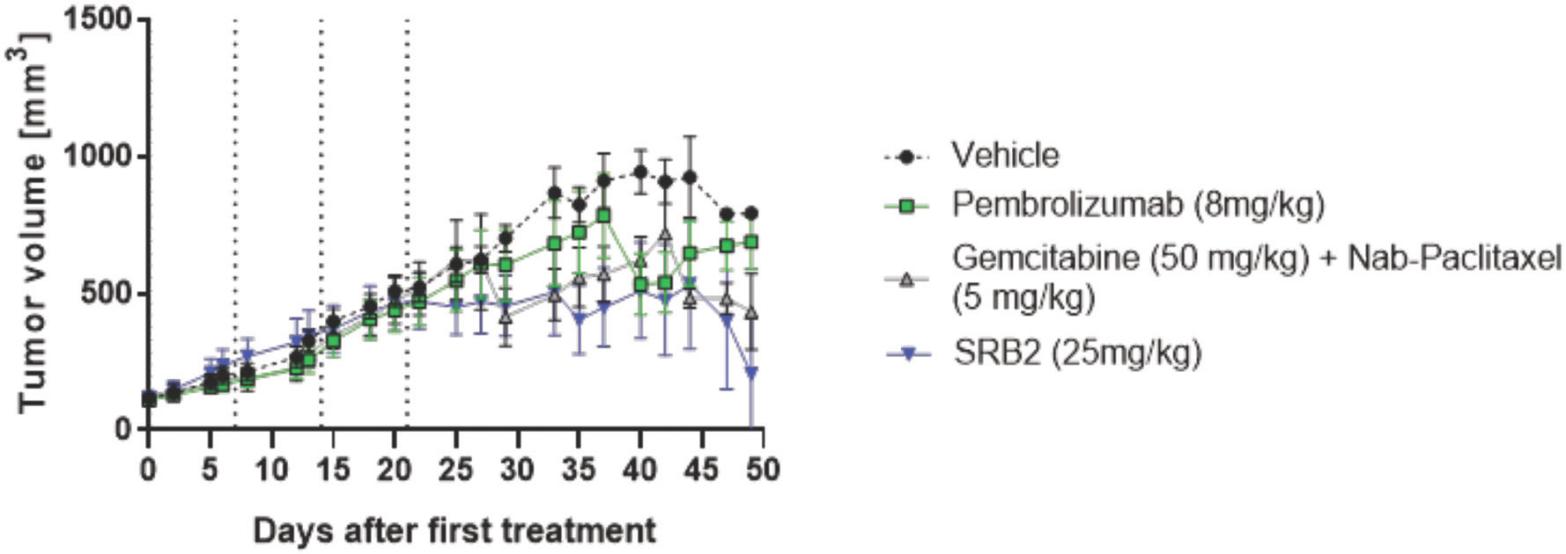
Tumor growth curves in NCG humanized mice. NCG mice with xenografted PDACs were treated with vehicle, SRB2 (25 mg/kg), pembrolizumab (8 mg/kg) or gemcitabine (50 mg/kg) + nab‐paclitaxel (5 mg/kg). Tumor volumes were lowest for SRB2 compared to vehicle (*P* = 0.031). Mean ± SEM for tumor volume for each treatment group were expressed in mm^3^ with *N* = 8 mice per group. Groups were compared with two‐way ANOVA.

We observed that SRB2 was safe when intravenously injected at 25 mg/kg in humanized mice. When assessing the comparison of multigroup treatment cohorts, mean tumor volume was not statistically different between groups, except for SRB2 compared to vehicle (*P* = 0.031) (Fig. 6). When examining individual treatment cohorts, SRB2 again had significantly improved antitumor efficacy compared to vehicle (*P* = 0.038) (Fig. S4). Notably, the SRB2 cohort had complete tumor regression for two mice, which were free of tumor at study completion. At study completion, tumors were well infiltrated by all types of human immune cells, including human leukocytes, CD3^+^ T cells, CD4^+^ T cells, CD8^+^ T cells, Tregs, NK cells, myeloid cells, and monocytes/macrophages (data not shown) demonstrating the ADCC‐driven mechanism of action of SRB2.

## 4 Discussion

The PDAC TME is composed of numerous cell types that coordinate signaling pathways to support cancer growth while suppressing immune function. The CCR9 chemokine axis was first described in thymocyte development and chemotaxis and has been of great interest in T‐cell maturation as it differentiates CD4 vs. CD8 lineage. However, reports now show that CCR9 is expressed on both cancer and stromal cells in the PDAC TME and regulates proliferation and invasion of PDAC cells. When both immunologic and oncologic putative roles of the CCR9/CCL25 axis are considered, it appears to be an ideal therapeutic target to directly kill cancer cells, to antagonize stromal fibroblasts in the PDAC TME, and to reactivate cytotoxic immune cells.

Failure of IO agents in PDAC has been attributed in part to the dense PDAC TME, but also to escape pathways from alternate signaling effectors. Here, our results suggest that SRB2 (anti‐CCR9 mAb) may help overcome immune resistance by directly targeting the candidate immune checkpoint CCR9 and by inhibiting the release of CCL25 (the ligand to CCR9). SRB2 functions as an Fc‐engineered humanized anti‐CCR9 mAb that increases Fc‐effector function to elicit an enhanced effector response. Beyond producing cell death through ADCC, SRB2 has the potential to block receptor‐ligand signaling to subsequently inhibit CCL25‐mediated proliferation and invasion of PDAC cells. Therefore, targeting CCR9 may improve the efficacy of current ICIs by blocking an alternate immune evasion pathway. Importantly, our study demonstrated the potential therapeutic applications of SRB2 in CCR9^+^ PDACs.

To accurately assess SRB2 efficacy, immune‐competent models were prerequisite for our investigational studies. Indeed, cytotoxic effects were observed in PDOs and PDXs containing CD3^+^ and CD45^+^ cell populations, but not in PDAC cell lines. In fact, we demonstrated that PDAC PDOs not only include cancer cells but also immune and stromal cells in early passage PDOs. Notably, we demonstrated that results for combination drug testing were similar between PDO and PDX models. While PDO and PDX models both recapitulate the PDAC TME, time to model formation is quicker and costs are lower when using PDOs [17]. Importantly, our study results demonstrated statistical significance in cytotoxic activity using SRB2 alone when compared to vehicle alone. Demonstration of positive activity in SRB2 therapy from pilot studies evaluating numerous treatment arms is the foundation for more focused, powered studies.

Standard‐of‐care systemic therapies for all PDAC patients have been FOLFIRINOX or gemcitabine/nab‐paclitaxel regimens [12]. Unfortunately, PDAC patients with unresectable disease have < 1 year median survival, and patients who undergo surgical resection of disease and receive perioperative chemotherapy have 5‐year survival rates around 20% [18, 19, 20]. As such, the lack of immunotherapeutic options has been glaring and reflects the failure of past approaches to incorporate IO agents. Yet, it is clear that more effective therapeutic strategies to prolong PDAC survival will require rational incorporation of immune‐based therapies. Our current study provides the first report of a novel humanized anti‐CCR9 mAb and its therapeutic activity in targeting PDAC. Given *in vitro* and *in vivo* activity, SRB2 shows great potential for multimodal therapy in PDAC.

## Conflict of interest

The authors disclose that DRL, EAC, PG, and LS are employees of SunRock Biopharma and have a financial interest in SRB2 (anti‐CCR9 monoclonal antibody). Other authors declare no conflict of interest.

## Author contributions

HGM, AMR, CB, MG, MG, AS, and MH wrote the manuscript, performed assays, revised, and edited the manuscript. MC, PKP, MBH, and JK developed the concept and revised and edited the manuscript. JR performed pathologic analysis of specimens and revised and edited the manuscript. AT, AT, and AV performed *in vivo* tests and revised and edited the manuscript. DRL, EAC, PG, and LS developed SRB2, performed *in vitro* tests, and revised and edited the manuscript.

## Peer review

The peer review history for this article is available at https://www.webofscience.com/api/gateway/wos/peer‐review/10.1002/1878‐0261.70062.

## Supporting information


**Fig S1.** CCR9 expression in PDAC cells and tissues.

**Fig S2.** Flow cytometry histograms.

**Fig S3.** CCR9 gene expression from The Cancer Genome Atlas.

**Fig S4.** Individual tumor growth curves in NCG humanized mice.

## Data Availability

The data generated in this study are available within the article and its supplementary data files. Any data not shown is available upon request from the corresponding author.
