# Supplementary figures and images for: Therapeutic applications of a novel humanized monoclonal antibody targeting chemokine receptor CCR9 in pancreatic cancer
Source: Mol Oncol. 2025 May 28;19(10):2978–88. doi: 10.1002/1878-0261.70062 (PMC12515709; doi:10.1002/1878-0261.70062)

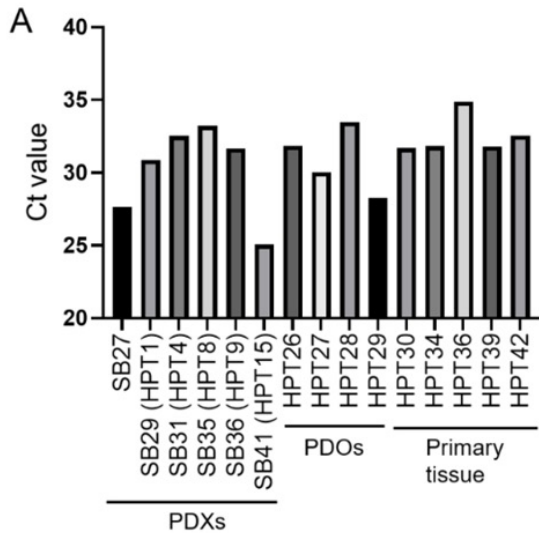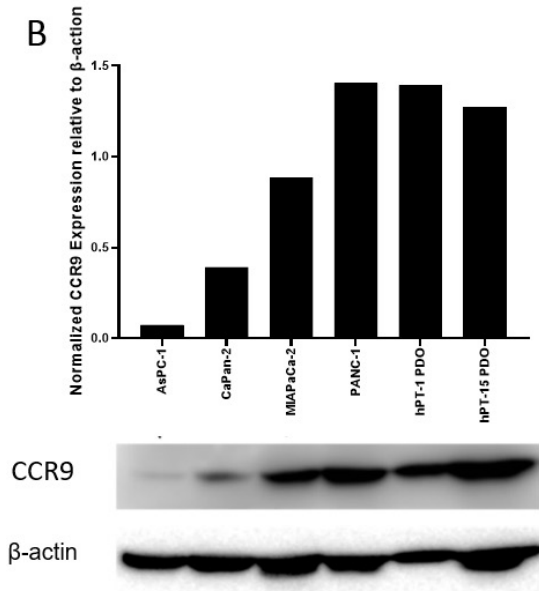

Supplement: Supplementary file 1 — Fig S1. CCR9 expression in PDAC cells and tissues. [file MOL2-19-2978-s002.pdf]

A

CAPAN-2

AsPC-1

MiaPaCa-2

Panc-1

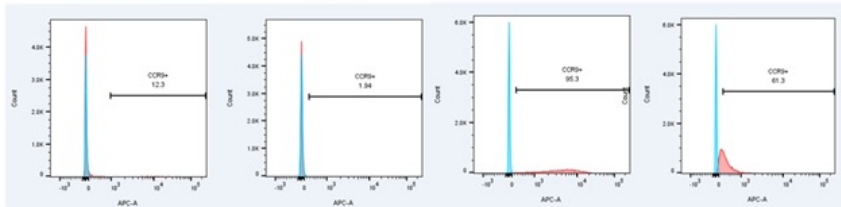

B

Unstained Control Cells

Stained Cells

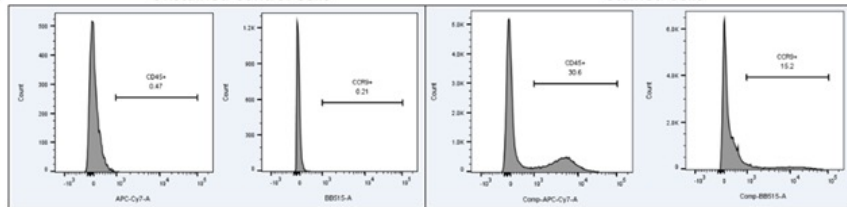

Supplement: Supplementary file 2 — Fig S2. Flow cytometry histograms. [file MOL2-19-2978-s003.pdf]

# PAAD Pancreatic adenocarcinoma

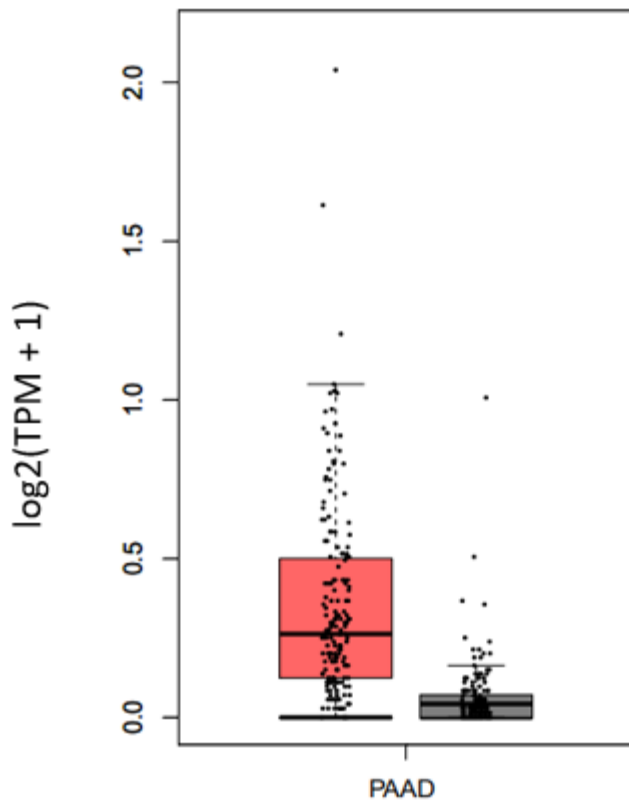

Supplement: Supplementary file 3 — Fig S3. CCR9 gene expression from The Cancer Genome Atlas. [file MOL2-19-2978-s001.pdf]

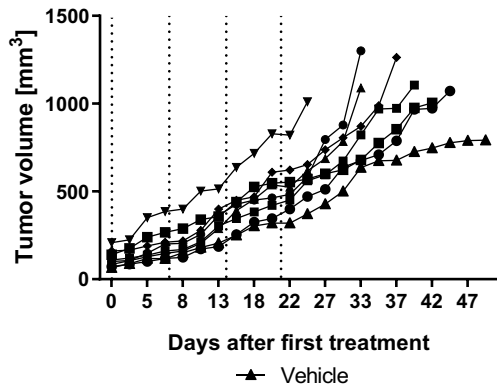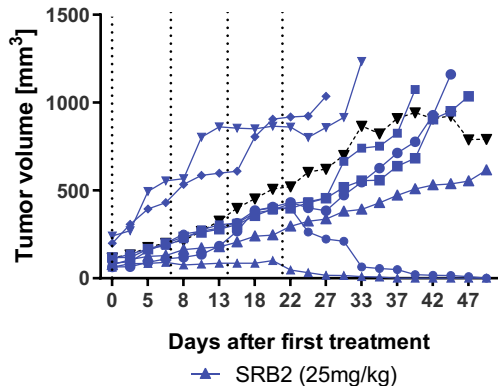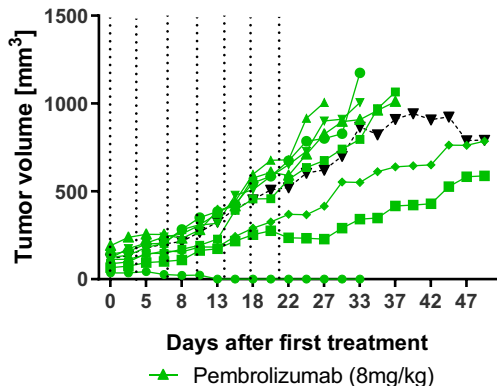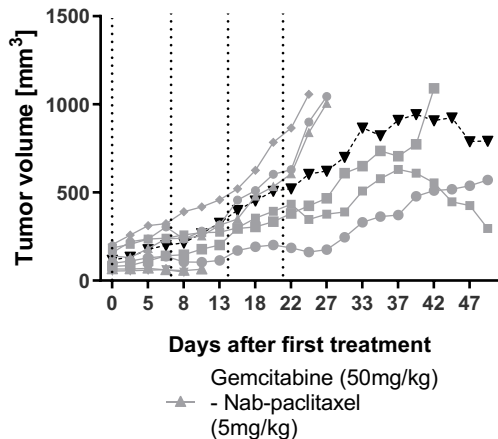

Supplement: Supplementary file 4 — Fig S4. Individual tumor growth curves in NCG humanized mice. [file MOL2-19-2978-s004.pdf]
